# Improved Cardiovascular Disease Outcomes in Older Adults

**DOI:** 10.12688/f1000research.7088.1

**Published:** 2016-01-28

**Authors:** Daniel E. Forman, Karen Alexander, Ralph G. Brindis, Anne B. Curtis, Mathew Maurer, Michael W. Rich, Laurence Sperling, Nanette K. Wenger

**Affiliations:** 1 Geriatric Cardiology Section, University of Pittsburgh Medical Center, Geriatric Research, Education, and Clinical Center, VA Pittsburgh Healthcare System, Pittsburgh, PA, USA; 2 Duke Clinical Research Institute, Duke University Medical Center, Durham, NC, USA; 3 Philip R. Lee Institute for Health Policy Studies, University of California, San Francisco, San Francisco, CA, USA; 4 Department of Medicine, University at Buffalo, Buffalo, New York, USA; 5 Department of Medicine, Columbia University Medical Center, New York, USA; 6 Washington University School of Medicine, St Louis, MO, USA; 7 Emory University School of Medicine and Rollins School of Public Health, Emory University, Atlanta, GA, USA; 8 Division of Cardiology, Department of Medicine, Emory University, Atlanta, GA, USA

**Keywords:** Geriatric, Cardiovascular, HFpEF

## Abstract

Longevity is increasing and the population of older adults is growing. The biology of aging is conducive to cardiovascular disease (CVD), such that prevalence of coronary artery disease, heart failure, valvular heart disease, arrhythmia and other disorders are increasing as more adults survive into old age.  Furthermore, CVD in older adults is distinctive, with management issues predictably complicated by multimorbidity, polypharmacy, frailty and other complexities of care that increase management risks (e.g., bleeding, falls, and rehospitalization) and uncertainty of outcomes.  In this review, state-of-the-art advances in heart failure, acute coronary syndromes, transcatheter aortic valve replacement, atrial fibrillation, amyloidosis, and CVD prevention are discussed.  Conceptual benefits of treatments are considered in relation to the challenges and ambiguities inherent in their application to older patients.

## Introduction

The unprecedented growth of the elderly population throughout the world is evolving as a challenge for clinicians, governments, and health policy guidance. Older adults differ from those typically studied in clinical trials, efficacy and outcomes of care often remain ambiguous for older patients, and growing healthcare cost burdens for older adults are a related concern. In this review, we highlight some key advances associated with improved outcomes that have recently occurred in the management of older adults with cardiovascular disease.

## Demographics

The population of older adults is growing rapidly, particularly as longevity is also increasing. In the United States, the population aged 65 and over is projected to be 83.7 million by 2050, almost double the 43.1 million in 2012 ^1^. The numbers of those who are very old (≥85 years) is growing the most rapidly. On a global level, the population ≥85 years is projected to increase 151% between 2005 and 2030, compared to the increase of only 104% in the population aged ≥65 years and only 21% for the population under 65 ^2^. Similar growth of elderly populations is occurring in both developed and developing countries. Between 2012 and 2030, the percentages of adults older than 65 years are expected to increase 33.2% in Japan, 27.9% in Germany, and 25.5% in Italy ^3^. In China, the world’s most populous country of 1.4 billion, the subgroup aged ≥65 years is expected to increase by 17.2% by 2030 and 26.8% by 2050, or stated as numbers, 350 million Chinese (a magnitude that is greater than the total US population) will soon be senior adults ^3^.

## Distinctive implications of aging

Geriatric features often transform healthcare challenges and are important concerns for clinical decision-making. Whereas cardiovascular management standards are fundamentally oriented to specific cardiac diseases, older adults typically present with multiple disease states occurring concurrently such that presentation and management of the cardiac issues are inherently more complex ^4^. In some cases, multimorbid disease states lead to compounding instabilities (e.g. heart failure [HF] and renal failure). In other cases, multimorbidities give rise to new disease states (e.g. HF and preserved ejection fraction [HFpEF] arising from the substrate of metabolic disease and inflammation) ^5^. Non-cardiac comorbid conditions also underlie much of the morbidity and rehospitalizations associated with HF, particularly HFpEF ^6^. Geriatric syndromes are part of multimorbidity; frailty, sarcopenia, cognitive decline, and other age-related dimensions of health fundamentally compound multimorbidity management complexities of cardiac conditions ^7^. These insights highlight the importance of a holistic approach in relation to HF as well as other cardiovascular diseases (CVDs) and call attention to the wide range of idiosyncratic capacities, vulnerabilities, and therapeutic objectives that typically distinguish one older cardiac patient from another. Similarly, typical cardiac medications (e.g. angiotensin-converting enzyme [ACE] inhibitors) must be regarded not only in terms of their evidence-based cardiac benefits but also in respect to the iatrogenesis they more easily (even predictably) provoke among older adults (e.g. syncope and falls) in the context of hypotension, balance or vision impairment, sleep deprivation, alcohol use, and other common conditions in an older patient population ^8^.

## Heart failure

Therapeutic options for the management of HF in older adults include medications, devices, and behavioral interventions. The PARADIGM-HF trial randomized 8442 patients with symptomatic HF and a left ventricular ejection fraction ≤40% to the ACE inhibitor enalapril or to the combination of the angiotensin-receptor blocker valsartan and the neprilysin inhibitor sacubitril ^9^. Compared to enalapril, valsartan/sacubitril was associated with significant reductions in cardiovascular death or hospitalization for HF, as well as all-cause mortality, cardiovascular mortality, HF hospitalizations, and HF-related symptoms. Patients ≥65 years and those ≥75 years derived similar benefits from combination therapy, as did younger patients. Based on these findings, it is anticipated that valsartan/sacubitril will be rapidly incorporated into the management of older and younger patients with HF and reduced ejection fraction (HFrEF).

In contrast to HFrEF, the treatment of patients with HFpEF, the vast majority of whom are elderly, remains problematic. In the Treatment of Preserved Cardiac Function Heart Failure With an Aldosterone Antagonist (TOPCAT) trial, spironolactone failed to reduce the primary outcome of cardiovascular death, aborted cardiac arrest, or HF hospitalizations ^10^. Spironolactone reduced HF hospitalizations at the expense of increased rates of hyperkalemia and elevated serum creatinine. Hence, the role of spironolactone in the management of HFpEF remains uncertain.

In the realm of devices, the value of implantable cardioverter-defibrillators (ICDs) in patients ≥75 years of age remains controversial, and selection of older patients for ICDs must be individualized ^11^. Mechanical circulatory support (MCS) appears to be associated with improved outcomes in carefully selected older adults with advanced HF, albeit with increased risk of complications compared to younger patients ^12^. Pre-morbid frailty is a strong predictor of worse outcomes in older adults receiving MCS ^13^.

In the domain of behavioral interventions, there is a need for novel approaches that address HF not in isolation, but rather in the context of a complex array of comorbid conditions and heterogeneous personal preferences regarding goals of care in order to optimize patient-centered outcomes ^14^. In addition, the management of HF in long-term care facilities and at the end-of-life now raises special considerations to maximize quality of life and reduce suffering ^15, 16^.

## Acute coronary syndrome

Recent developments in antithrombotic therapy as well as better information on frailty and outcomes have benefitted older adults with acute coronary syndrome (ACS). A clinical challenge in older adults presenting with a myocardial infarction (MI) or undergoing percutaneous coronary intervention (PCI) is the indication for multiple antithrombotic agents (e.g. atrial fibrillation [AF] or valvular disease). Triple therapy increases the rate of major bleeding, an outcome already common among older adults. Two recent European registry studies further the understanding of antithrombotic therapy in patients with AF with MI and/or undergoing PCI. Among older adults requiring oral anticoagulation presenting with MI or undergoing PCI, clopidogrel added to oral anticoagulant therapy was as effective as triple therapy (including aspirin) in preventing MI/coronary death, ischemic stroke, and bleeding ^17^. In another study, among patients requiring oral anticoagulation undergoing PCI (80% ACS, 65% drug-eluting stent and 30% bare metal stent), use of clopidogrel without aspirin was also associated with less bleeding and no increase in thrombotic events during follow up ^18^. A supporting secondary analysis from a large AF clinical trial found the oral anticoagulant apixaban had similar beneficial effects on stroke, MI, and major bleeding, with or without aspirin, compared with warfarin ^19^. Ongoing research is exploring the ability to omit aspirin in the setting of an effective oral anticoagulant. These new and safer antithrombotic strategies will be an important advance for high-risk individuals with coronary artery disease and indications for oral anticoagulation.

Older adults are at high risk of adverse outcomes following MI, but a Medicare linkage analysis from the CRUSADE Registry provides evidence that most mortality occurs early and not in the context of rehospitalization. Contrary to expectations, rehospitalization rate did not rise substantially with age, in part due to competing mortality. Rehospitalizations which did occur were often for non-cardiac diagnoses, underscoring the multimorbidity also present in the older population with a coronary event ^20^. The TaRgeted platelet Inhibition to cLarify the Optimal strateGy to medicallY manage Acute Coronary Syndromes (TRILOGY ACS) trial, which randomized 9326 patients with MI planned for medical management to prasugrel or clopidogrel, added frailty assessment at baseline for patients aged ≥65 years ^21^. The modified Fried score classified 23.0% of the older adults in this trial as pre-frail (1–2 items) and 4.7% as frail (≥3 items). After adjustment, frailty remained independently associated with the composite of cardiovascular death, MI, or stroke: pre-frail vs. not-frail, hazard ratio (HR): 1.33; 95% confidence interval (CI): 1.15–1.54; p<0.001; frail vs. not-frail, HR: 1.52; 95% CI: 1.18–1.98; p=0.002. Frailty among registry populations is higher than in clinical trials, so care delivery models should add early targeted follow up in older adults after MI, particularly for those with multimorbidity or frailty. Future studies will benefit from collecting frailty data to allow comparative effectiveness and outcomes comparisons across older MI or PCI populations.

## Transcatheter aortic valve replacement

Transcatheter aortic valve replacement (TAVR) represents an innovative interventional technology that provides a non-surgical alternative for the management of severe aortic stenosis (AS) that has particular relevance for the elderly and the very elderly populations. The prevalence of AS in the US is estimated to be greater than 4–5% of those over 75 years of age. Approximately 50% of all US patients and over 75% of those over 80 years of age having clinical indications for surgical aortic valve replacement (AVR) are not being surgically treated due to either the lack of referral by clinicians or patient/family refusal. The use of TAVR has been projected to make substantial inroads in this underserved population, particularly those who are very elderly or deemed at high surgical risk.

The key randomized clinical trials (RCTs) PARTNER A & B (utilizing Edwards’ Sapien balloon expandable AVR) along with the CoreValve (Medtronic’s self-expanding percutaneous AVR) study of high-risk patients conclusively demonstrated improved long-term patient survival employing TAVR compared to sAVR (standard surgical AVR) with acceptable stroke and bleeding rates for patients felt either surgically inoperable or at very high surgical risk. The median age of TAVR implantation in these study cohorts was approximately 84 years ^22, 23^.

TAVR had been available for clinical use in over 31 countries prior to United States FDA approval in 2011. To help ensure a rational diffusion of TAVR’s innovative technology partnerships between the Food and Drug Administration (FDA), the Center for Medicare and Medical Services (CMS) along with cardiovascular professional societies led by the American College of Cardiology (ACC) and the Society for Thoracic Surgery (STS) collaborated in the creation of critical Post Approval Studies, CMS Coverage Determination Criteria, a multi-societal competency document for institutional and professional utilization of TAVR technology accompanied by an expert consensus document and clinical practice guidelines for TAVR clinical use. STS and ACC have created the STS/ACC transcatheter valve therapy (TVT) Registry where all commercial TAVR implants are required to be enrolled by hospitals to satisfy CMS coverage and payment requirements ^24– 27^. As of spring 2015, over 30,000 TAVR patients from approximately 350 institutions have been entered into the TVT Registry.

The TVT Registry data has been instrumental in assuring quality of care along with providing the infrastructure for performing Post Approval Studies, Investigational Device Exemption Studies, and Post Market Surveillance ^28, 29^. The Registry allowed earlier FDA approval than would be possible through previous RCT mechanisms for “Valve-in-Valve” TAVR use and also for alternative access use in high-risk and surgically inoperable patients. The TVT Registry demonstrated 7.0% 30-day mortality, and 23.7% 1-year mortality with a stroke rate of 4.1% ^30^.

Of great importance for assessing clinical decision-making for elderly patients with AS, the TVT Registry is collecting both baseline and longitudinal data on frailty and activity limitation assessments through 5-meter walk tests and the Kansas City Cardiomyopathy Questionnaire (KCCQ). In addition, the TVT Registry is creating a risk-adjusted mortality model that will hopefully soon be transformed into a predictive outcome tool to help clinicians, patients, and families make informed decisions in the management of elderly and very elderly patients with AS.

## Atrial fibrillation

The median age of patients with AF is 75 years, with a prevalence of about 9% in the elderly ^31^. Stroke stands out as one of the greatest risks attributable to this arrhythmia. The 2014 American Heart Association/ACC/Heart Rhythm Society (AHA/ACC/HRS) guideline ^32^ recommends using the CHA _2_DS _2_-VASc (Congestive HF [CHF], Hypertension, Age ≥75 years, Diabetes, Stroke/Transient ischemic attack [TIA], Vascular disease, Age 65–74, Sex category [female]) score to assess stroke risk. The guideline recommends oral anticoagulant therapy for AF patients with a CHA _2_DS _2_-VASc >2 using warfarin or one of the new oral anticoagulants (NOACs) dabigatran, rivaroxaban, or apixaban. Edoxaban is another NOAC that was approved for use after the guideline was published, but it has efficacy that is comparable to those approved earlier.

As age >75 years leads to a minimum CHA _2_DS _2_-VASc score of 2, many emphasize the value of anticoagulation for most elderly patients with AF and decry the underuse of such vital therapy. Nonetheless, this still remains an issue of debate amidst age-related intricacies of care, i.e. intrinsic bleeding risks are often compounded by frailty, multimorbidity, polypharmacy, falls, and other management complexities.

There have been several studies to estimate the risk/benefit ratio of treating elderly AF patients with oral anticoagulation, including the Birmingham Atrial Fibrillation Treatment of the Aged (BAFTA) study ^33^, which randomized individuals aged ≥75 years of age to aspirin or warfarin based on physician discretion. Stroke/systemic embolism (SE) occurred at a rate of 1.8%/year in warfarin-treated patients versus 3.8%/year in the aspirin group (HR 0.48; 95% CI, 0.28–0.80), with no difference in the rates of major hemorrhage (1.9 vs. 2.2%, respectively). The risk for bleeding with warfarin therapy in patients >80 years of age has been estimated to range from 1.63% to 13.1% per year ^34, 35^.

The NOACs have generated much excitement in the field of AF management, as they enable relatively simplified treatment compared to warfarin (e.g. reduced need for blood testing and no dietary restrictions). NOACs that include both direct thrombin inhibitors (dabigatran) and factor X inhibitors (rivaroxaban, apixaban, and edoxaban) are now available as alternatives to warfarin. All have been shown to be non-inferior to warfarin with respect to stroke and SE, with comparable rates of bleeding ^36– 39^. The mean age of patients in these studies was approximately 70 years, with about one-third of the patients being over 75 years.

A recent meta-analysis of the efficacy and harm of the NOACs for prevention of stroke in AF and secondary prevention of thromboembolism found that the efficacy of each of the NOACs was equal or superior to warfarin in elderly patients ^40^. Dabigatran was associated with a higher risk of gastrointestinal bleeding and a lower risk of intracranial bleeding. A significantly lower risk of major bleeding compared to warfarin was found for apixaban (odds ratio 0.63, 95% CI 0.51–0.77) and edoxaban 60 mg (0.81, 0.67–0.98) and 30 mg (0.46, 0.38–0.57), while rivaroxaban showed similar risks.

## Amyloidosis

Cardiac amyloid has traditionally been considered a rare cardiovascular condition. However, emerging data demonstrate that transthyretin cardiac amyloidosis (ATTR), formerly known as senile cardiac amyloidosis, caused by misfolded monomers or oligomers of the protein transthyretin (TTR), is a common cause of HFpEF. Indeed recent autopsy studies in HFpEF subjects (mean age 76 years) showed 21% had amyloid deposits and the prevalence was greater in those ≥75 years (32%) vs. <75 years (8%). Furthermore, only 20% of the HFpEF patients with amyloid at autopsy had received a pre-morbid diagnosis ^41^.

Bone isotopes, commonly used in bone scans, have a high sensitivity and specificity (>90%) for identifying TTR cardiac amyloid due to either wild-type TTR or a mutant allele ^42, 43^. While over 20 mutations can cause TTR cardiac amyloidosis, the Val122Ile mutation (substitution of isoleucine for valine at position 122) is the most common in the United States with a particularly high frequency (prevalence of 3.4% to 3.9%) in African-Americans ^44^. The Thr60Ala mutation (substitution of alanine for threonine at position 60) is also notably common in individuals of Irish descent and is referred to as the Appalachian mutation. These genetic conditions have an age-dependent penetrance, with males over the age of 60 years being most commonly affected.

Historically, cardiac amyloid was difficult to diagnose because it can masquerade as other cardiovascular disorders ^45^ and usually required endomyocardial biopsy. The diagnosis has become easier with the discovery that bone isotopes have a very high sensitivity and specificity for distinguishing ATTR cardiac amyloid (both mutant and wild-type) from light chain (AL) cardiac amyloid ^46^ and other types of cardiomyopathy that mimic amyloid (e.g. hypertrophic cardiomyopathy). Differentiating TTR from AL cardiac amyloid has important prognostic, management, counseling, and therapeutic implications. These nuclear medicine techniques have the potential to dramatically alter the outcomes of patients with ATTR cardiac amyloidosis, particularly because new therapeutic options are looming.

Emerging therapeutic strategies to treat ATTR amyloid are focused on small molecules to stabilize the transthyretin tetramer and agents to silence TTR production. Phase II clinical trial data ^47^ suggest that tafamidis was generally well tolerated and stabilized TTR, leading to a phase III clinical trial that has completed enrollment. TTR silencers using either small interfering RNA (siRNA) or oligonucleotides specific for silencing TTR production have shown remarkable ability to lower TTR to >80% of normal levels in subjects with TTR cardiac amyloidosis. Collectively, such data remind us that TTR cardiac amyloid is certainly not rare and hopefully not unmodifiable.

## Preventive care

Recent guidelines for cardiovascular prevention importantly emphasize that older adults are at highest risk for atherosclerotic cardiovascular events. Paradoxically, however, strategies focused on cardiovascular prevention are often underutilized in this subpopulation ^48^. As the elderly also have the greatest burden of comorbidities, polypharmacy, and potential for medication-related side effects, the need for comprehensive and collaborative clinician-patient management is imperative ^49^.

Potential overtreatment of hypertension, in addition to inadequate control, may cause adverse outcomes in the elderly, who are more likely to have target organ damage. Considerable debate has arisen from the guideline recommendations made by the panel members appointed to JNC 8 (Eighth Joint National Committee) that blood pressure should be reduced to less than 150/90 mm Hg in adults aged 60 years and older without diabetes or chronic kidney disease ^50^. In support of the ACC/AHA 2011 Expert Consensus document on hypertension in the elderly ^51^ that recommended blood pressure be reduced to less than 140/90 in adults aged 60–79 (and a systolic pressure of 140–145 if tolerated in adults aged 80 and older) are data from REGARDS (REasons for Geographic and Racial Differences in Stroke), which reported that optimal blood pressure for elderly patients on antihypertensive therapy for the reduction of cardiovascular events and all-cause mortality was less than 140/90 mm Hg ^52^.

Several recommended caveats for the care of older adults are highlighted by the ACC/AHA 2013 guidelines for the treatment of blood cholesterol, including the use of moderate-intensity statin therapy in adults >75 years of age with established atherosclerotic CVD (ASCVD). The ASCVD pooled cohort risk estimator does not provide for risk assessment in patients >79 years of age ^53^. When initiating statin therapy, the risk of transitioning to diabetes should be discussed, although this risk is small in relation to the potential benefits of statin medications. Importantly, there is no definitive evidence that statins lead to cognitive decline. The available data on the use of proprotein convertase subtilisin/kexin type 9 (PCSK9) inhibitor therapy in those of advanced age is promising, as low-density lipoprotein (LDL)-C is potently reduced with a side effect profile similar to that of placebo ^54^.

Lifestyle approaches remain the cornerstone for cardiovascular prevention in the elderly. Among persons aged 55 to 80 (mean age 67) at increased cardiovascular risk, a Mediterranean diet supplemented with extra-virgin olive oil or mixed nuts reduced the incidence of major cardiovascular events by 30% ^55^. Emphasizing a goal of living longer and better, a Mediterranean lifestyle including dietary pattern, moderate alcohol intake, regular physical activity, and not smoking was associated with a 60% reduction in all-cause mortality in elderly Europeans aged 70–90 ^56^.

## The value of geriatric measures in patient-centered decision-making for clinical cardiovascular problems

In general, medications, procedures, and other components of cardiovascular care are more likely to entail trade-offs at elderly age. Polypharmacy, pain, fatigue, confusion, dysgeusia, excessive time for recovery, and other sequelae may detract from (and sometimes even supersede) intended therapeutic benefits ^57, 58^. Whereas life prolongation is the overriding goal of therapy in the young, for many seniors issues of independence, quality of life, and functional capacity are often more important, and this may determine therapeutic choices that contrast with those of younger populations. This broader spectrum of clinical objectives and the high susceptibility to iatrogenesis are driving rationales for a growing emphasis on principles of shared decision-making between older patients and their clinicians ^57, 58^.

Other age-related issues that commonly affect caregiving of older cardiac patients include frailty, altered body composition (diminished lean body mass and increased interstitial fat), and changes in cognition. Frailty is often assessed as a constellation of phenotypic changes that relate to a state of increased vulnerability, i.e. slowing, weakening, weight loss, exhaustion, and reduced activity ^59^. Patients with three or more of these measures are considered frail and are generally at increased risk for disease but also for poor tolerance of therapy. Frailty therefore fundamentally factors into therapeutic decisions and management strategies. Sarcopenia or reduced lean body mass is a subpart of frailty and directly detracts from physical capacity and vital resiliency to recover ^60^. Diminished cognition, particularly executive function, is similarly insidious and detrimental, and complicating with regard to decision-making and adherence, and even in respect to the quality of life benefits that are achievable by therapy ^61^.
